# A Japanese hereditary spastic paraplegia family with a rare nonsynonymous variant in the *SPAST* gene

**DOI:** 10.1038/s41439-021-00153-x

**Published:** 2021-05-25

**Authors:** Takuya Morikawa, Shiroh Miura, Takahisa Tateishi, Kazuhito Noda, Hiroki Shibata

**Affiliations:** 1 Division of Genomics, Medical Institute of Bioregulation, Kyushu University, 3-1-1, Maidashi, Higashi-ku, Fukuoka, Japan; 2 Department of Neurology and Geriatric Medicine, Ehime University Graduate School of Medicine, Shitsukawa, Toon, Ehime Japan; 3 Division of Respirology, Neurology, and Rheumatology, Department of Medicine, Kurume University School of Medicine, 67 Asahimachi, Kurume, Japan; 4 Nodakousei Clinic, 5-4, Kurihara, Ogi, Japan

**Keywords:** Neurodegeneration, Neurodegeneration

## Abstract

Spastic paraplegia (SPG) type 4 is an autosomal dominant SPG caused by functional variants in the *SPAST* gene. We examined a Japanese family with three autosomal dominant SPG patients. These patients presented with typical symptoms of SPG, such as spasticity of the lower limbs. We identified a rare nonsynonymous variant, NM_014946.4:c.1252G>A [p.Glu418Lys], in all three family members. This variant has previously been reported in a Russian SPG family as a “likely pathogenic” variant.^5^ Ascertainment of additional patients carrying this variant in an unrelated Japanese SPG family further supports its pathogenicity. Molecular diagnosis of SPG4 in this family with hereditary spastic paraplegia is confirmed.

Hereditary spastic paraplegia (HSP) is a group of clinically and genetically heterogeneous disorders characterized by progressive lower-limb spasticity and weakness as a result of corticospinal dysfunction^1,2^. The most common type of HSP is spastic paraplegia 4 (SPG4, MIM: 182601), accounting for 15–40% of all HSP cases^3^. SPG4 is autosomal dominant and caused by functional variants in the *SPAST* gene, which is located at 2p22.3 and encodes the microtubule-severing protein spastin^1,4^.

Kadnikova et al.^5^ previously identified NM_014946.4:c.1252G>A [p.Glu418Lys] in a single Russian spastic paraplegia (SPG) family^5^. Although the authors reported the variant as “likely pathogenic” according to the guidelines of the American College of Medical Genetics (ACMG), the Association for Molecular Pathology (AMP), and the College of American Pathologists (CAP)^5,6^, it is necessary to assess additional patients carrying the same variant in unrelated SPG families for a final conclusion regarding the pathogenicity of the variant.

We evaluated a Japanese pedigree of SPG including three patients (proband (I-2), her daughter (II-2), and son (II-3)) (Fig. 1). The proband was a 55-year-old Japanese woman who noticed a stretched feeling of the lower limbs approximately at age 40. She needed a cane for walking. On neurological examination, she showed typical SPG, with a moderate vibration sense decrease in the lower extremities. The proband’s daughter (II-2) was a 30-year-old who had a history of congenital cataracts in her right eye. Although she had no subjective symptoms, she exhibited hyperreflexia and spasticity in the lower extremities. The proband’s son (II-3) was a 26-year-old man who noticed a stretched feeling of the lower limbs at age 12; at age 21, he could not move down stairs without a handrail. He also showed typical SPG, with slightly impaired vibration sense in the lower extremities.

**Fig. 1:**
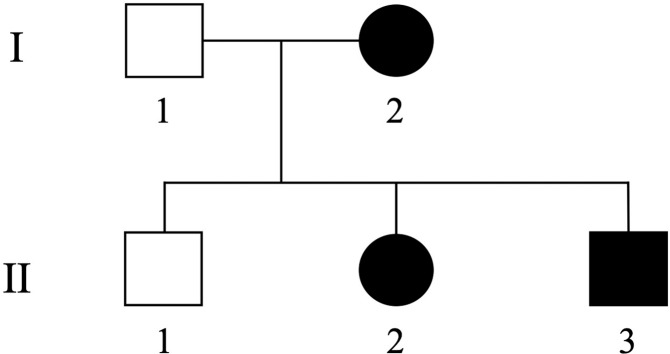
Pedigree of the tested family. Squares: males, circles: females, solid symbols: affected individuals, open symbols: unaffected individuals.

Whole-exome sequencing was performed for the three affected family members, I-2, II-1 and II-2, and one unaffected family member, II-3 (Fig. 1). We selected functional variants shared by the three patients but not by the unaffected individual. Missense, nonsense, frameshift, insertion/deletion, and splice site variants are considered functional variants. A summary of the exome sequencing results is shown in Supplementary Table S1. We identified 1331 variants shared by the three patients but the unaffected individual. Using the information of 79 genes reported to be responsible for SPG^1^, we identified seven variants in six genes, of which two in two genes are nonsynonymous. Among these, one variant showed a minor allele frequency <0.02% in both 1000G project and ExAc: NM_014946.4:c.1252G>A [p.Glu418Lys]) in exon 10 of the *SPAST* gene. This variant has been previously reported by Kadnikova et al.^5^ as a “likely pathogenic” variant in one Russian SPG family^5^. By using PCR and Sanger sequencing for all family members (forward primer, 5′-ACTCTCCCCTTTCTCAAACCA-3′ and reverse primer, 5′-AGTCTTTAAGCTTGCCCTTCT-3′), we confirmed cosegregation of the variant with the disease in the pedigree (Fig. 2).

**Fig. 2:**
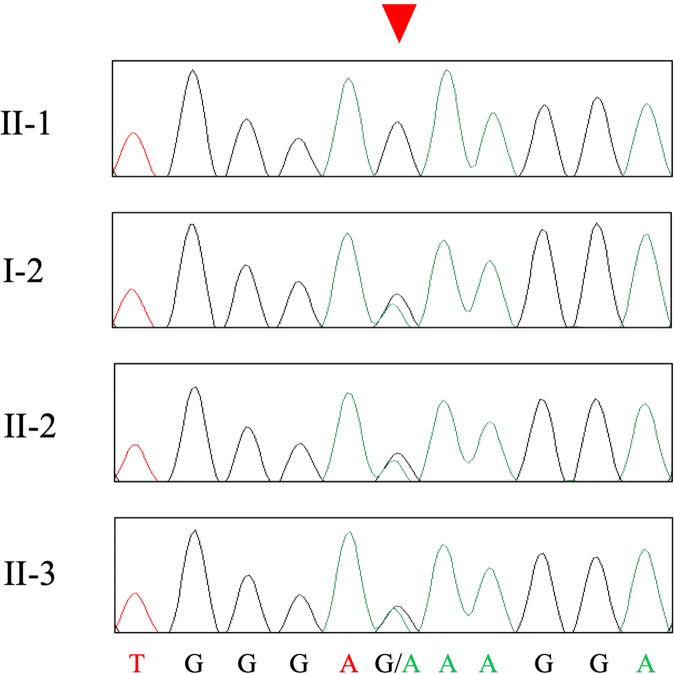
Electropherogram of the region of the variant NM_014946.4:c.1252G>A [p.Glu418Lys] in one unaffected (II-1) and three affected (I-2, II-2 and II-3) family members. The location of the variant is indicated by a red triangle.

The variant is predicted to be “damaging” by Polyphen-2. A high CADD score that was marginally significant (CADD = 34) was also observed for the variant. This variant is located in a conserved ATPase domain of spastin. Several variants located in the domain have been reported to be responsible for SPG4 (refs. ^7–10^). The variant has been previously reported from a Russian SPG family; it was found in a Japanese SPG family in the current study^5^. By combining the two studies, the variant meets the criteria of PM1, PM2, PM5, PP1, PP2, and PP3 of the ACMG/AMP/CAP guideline, and its pathogenicity is further supported. Therefore, we conclude that NM_014946.4:c.1252G>A [p.Glu418Lys] is highly likely to be a causative variant for SPG4.

## Supplementary information

Supplementary Table 1

## Data Availability

The relevant data from this Data Report are hosted at Human Genome Variation Database at 10.6084/m9.figshare.hgv.3015.
