# Supplementary material for: A Japanese hereditary spastic paraplegia family with a rare nonsynonymous variant in the SPAST gene
Source: Hum Genome Var. 2021 May 25;8:21. doi: 10.1038/s41439-021-00153-x (PMC8149642; doi:10.1038/s41439-021-00153-x)
Supplement: Supplementary file 1 — Supplementary Table 1 [file 41439_2021_153_MOESM1_ESM.pdf]

Supplementary Table 1. Summary of exome sequencing.

	I-2	II-1	II-2	II-3
Number of reads	18,983,289	45,505,136	19,439,518	25,500,440
Number of bases	1,442,729,964	6,825,770,400	1,477,403,368	1,938,033,440
Median of read depth	28.9	136.5	29.5	38.8
Total variants	111,829	121,656	20,190	136,816
